# Patterns Recognized in Ayres Sensory Integration^®^: A Scoping Review

**DOI:** 10.3390/brainsci16070700

**Published:** 2026-06-30

**Authors:** Marica Botha, Janine van der Linde, Zoe Mailloux

**Affiliations:** 1 Department of Occupational Therapy, Faculty of Health Sciences, University of the Witwatersrand, Johannesburg 2191, South Africa; janine.vanderlinde@wits.ac.za; 2 Department of Occupational Therapy, Thomas Jefferson University, Philadelphia, PA 19107, USA; zoemailloux@gmail.com; 3 Collaborative for Leadership in Ayres Sensory Integration (CLASI), Redondo Beach, CA 90277, USA

**Keywords:** sensory integration, sensory integration dysfunction, sensory processing disorder, pediatric neurodevelopment, occupational therapy assessment, psychometric assessment, child neurodevelopment, evidence synthesis

## Abstract

**Highlights:**

**What are the main findings?**

- Test interpretation in Ayres Sensory Integration^®^ is based on 60 years of research on sensory integration patterns.
- Sensory integration patterns show similarities, yet some differences were found across locations and diagnostic populations.

**What are the implications of the main findings?**

- Sensory integration patterns exhibit variation in new studies; therefore, Ayres Sensory Integration^®^ (ASI) practitioner should stay up to date with ongoing research.
- This study provides a comprehensive overview of sensory integration patterns in ASI, confirming it is grounded in rigorous research.

**Abstract:**

**Background/Objectives**: Associations between scores on tests of sensory integration functions demonstrated in numerous studies over six decades have contributed to the development of Ayres Sensory Integration^®^ (ASI) theory and practice. Understanding these “patterns” aids therapists in their interpretation of assessment results, ensuring appropriate and effective intervention. This study aimed to describe and discuss published research on identified patterns related to constructs in the Ayres Sensory Integration^®^ approach. **Methods**: Arksey and O’Malley’s framework and the Preferred Reporting Items for Systematic Reviews and Meta-Analyses Extension for Scoping Reviews Checklist were used. The literature review search was guided by the following criteria: population (children aged from 3 years to 12 years and 11 months), context (the literature within ASI), and concept (sensory integration patterns). Data were sourced from the following databases: CHINAL, ProQuest (Health & Medical Complete), SABINET, Web of Science, PubMed, OpenGreyEU, Greylit.org, and CADTH GreyMatters. There was no date constraint, and all available data were collected. **Results**: After reviewing 998 peer-reviewed articles, 17 articles met the inclusion criteria. Data extraction, charting, and analysis indicating a clear pattern of assessments were used to determine the sensory integration patterns. Similarities and differences between sensory integration patterns were found over time, in different diagnostic groups and different settings. **Conclusions**: The ASI approach is based on detailed research on the development of assessment instruments used for evaluation and sensory integration patterns used for interpretation of the assessment results. This research has been conducted over the past 60 years and contributes to an evidence-based therapeutic approach.

## 1. Introduction

When A. Jean Ayres began developing her theory of sensory integration, now referred to as Ayres Sensory Integration^®^ (ASI) [1], she conducted studies to better understand associations between sensory and motor functions. These studies resulted in the identification of the main constructs upon which the ASI approach is based. The associations between sensory and motor functions were determined through factor analysis (FA), and sometimes cluster analysis (CA), and lead to the concept of “patterns”. Some examples of the identified patterns include dyspraxia [2,3]; [S11,S12], bilateral integration and sequencing [2,3,4,5]; [S10–S12,S15], visio- and somatodyspraxia [4,5,6]; [S10,S13,S15], and visual perception [2,7,8]; [S11,S4,S17]. Identification of these patterns has been intended to assist therapists in recognizing known relationships between functions, rather than providing a descriptive label for individual children (i.e., the patterns are not intended as diagnoses or labels). Therapists with advanced training in ASI use knowledge about patterns identified through previous FA- and CA-based research to understand and verify the sensory integration abilities and challenges in children. Understanding of the patterns also justifies the basis for choosing a specific intervention approach, assists in goal setting and intervention planning, and contributes to appropriate therapeutic outcomes [6,9,10,11].

To understand sensory integration patterns and their differences, understanding of the two main quantitative statistical methodologies used to develop the patterns is essential. The first of these methodologies, FA, aims to simplify complex, interrelated variables to uncover latent structures that account for the relationships among a large set of variables [12,13,14]. By reducing a large correlation matrix to a smaller set of common factors, FA enables researchers to clarify the shared characteristics between variables [14]. It can be “exploratory,” when typically used early in research or instrument development to identify variables that cannot be assessed directly, or “confirmatory,” when used to verify factor structure from previous research [14]. Therefore, FA explains covariation among variables by identifying latent constructs; it aims to consider why variables may be correlated. The second methodology, CA, is used to identify naturally occurring subgroups of individuals who exhibit similar score patterns on a set of tests [3,13,15,16]. Unlike FA, which yields continuous dimensions, CA produces categorical groupings [13]. Thus, CA addresses the question of which individuals are most similar, assigning each individual to a single group.

Ayres’ theory—now referred to as ASI—encompasses theory, principles regarding efficient sensory integration underlying learning, assessment instruments, principles of intervention, a guide to manualize intervention, and a fidelity measure to direct practice and research [1,11,17,18]. Ayres drew extensively on the neuroscience literature to conceptualize previously unexplored sensory deficits—here understood as distinct sensory integration patterns—that influence both behavior and learning [19]. At present, neuroscience continues to constitute a foundational component of ASI [20,21]. Empirical investigations reviewed by Schaaf et al., Schoen et al., and Steinbrenner et al. demonstrated that ASI intervention provided to children with autism fulfills the established criteria for an evidence-based intervention [22,23,24].

As both a therapeutic practice and a field of study, ASI embodies a dedicated effort to meaningfully understand the human condition and improve understanding of how variations in neural functioning influence engagement and participation. Over the past six decades, advances in neuroscience have enabled current scientists to clarify and confirm sensory integration patterns developed from Ayres’ research [19].

Ayres first created individual tests [25,26,27,28,29], followed by a locally normed group of tests (the Southern California Sensory Integration Tests (SCSIT) [30] and, finally, a nationally normed group of tests (the Sensory Integration and Praxis Tests (SIPT) [4]. Between 1963 and 1989, she conducted FA and CA using various combinations of these tests, alongside non-standardized observations and questionnaires. These studies included samples from typically developing children, children with learning disabilities, and children with possible sensory integration difficulties [6]. From 1998 to 2015, additional studies were conducted to verify and clarify patterns in different populations and settings [2,3,5,6]; [S11,S12,S15,S16]. Since 2015, only one study related to sensory integration patterns has been published [8]; [S17].

Over the six decades of research, although the assessment instruments applied varied greatly between studies, consistent patterns emerged [5,6,11,31]. Limitations included limited availability of ASI assessment instruments, especially related to age range; global representation in the norms; and the cost of available assessments [5,6,31]; [S15,S13,S16]. These limitations led to the development of the Evaluation of Ayres Sensory Integration^®^ (EASI), introduced in 2018 [32]. The new set of tests constituting the EASI are in ongoing development.

To date, to the best of the authors’ knowledge, no scoping review examining sensory integration patterns has been published. Accordingly, the purpose of this scoping review was to identify and map the breadth of published evidence on sensory integration patterns of function and dysfunction in ASI. In addition, this review sought to identify gaps in the existing body of knowledge. Specifically, the aim was to describe and discuss current research on sensory integration patterns in ASI and identify areas that require further investigation. A comprehensive mapping of the existing literature on sensory integration patterns will provide a foundation for understanding these patterns.

## 2. Materials and Methods

This scoping review followed the updated methodological framework of Peters et al.: (1) define the title and review question; (2) define the eligibility criteria; (3) identify information sources; (4) define the search strategy; (5) identify and select relevant studies; (6) chart the data; and (7) present, summarize, and report the evidence [33]. To improve the structure and clarification for each step of the scoping review process, we used the Preferred Reporting Items for Systematic Reviews and Meta-Analyses Extension for Scoping Reviews (PRISMA-ScR) checklist (Table S1) [34].

### 2.1. Review Question

What is known from the literature regarding sensory integration patterns of function and dysfunction identified in the ASI approach?

### 2.2. Eligibility Criteria

The population, context, and concept (PCC) approach was used to develop the eligibility criteria for this scoping review. The population was defined as children aged 3 years to 12 years and 11 months, corresponding to the age range for which the performance-based assessment instruments within ASI have been standardized. The context was limited to the ASI literature; consequently, only studies linked to ASI and the theory developed by Ayres were included. Relevant articles were identified using the term “Ayres Sensory Integration^®^,” referring to theory and practice developed by Ayres, in conjunction with assessment instruments developed by her (e.g., the SCSIT or SIPT). The concept explored was sensory integration patterns. We chose not to limit the search by study design, location, or diagnosis; however, only articles published in English were included. Studies that did not include at least one performance-based assessment instrument based on ASI were also excluded, to ensure that sensory integration patterns were identified through standardized assessment instruments grounded in the ASI theory. We included studies that used additional parent-report questionnaires and assessment instruments not developed by Ayres, provided these were used in conjunction with at least one ASI performance-based assessment instrument.

### 2.3. Search Strategy

We carried out the searches between 23 August and 22 September 2023, using the following electronic databases: CHINAL, ProQuest (Health & Medical Complete), SABINET, Web of Science, PubMed, OpenGreyEU, Greylit.org, and CADTH GreyMatters. After the articles were selected and included, we conducted a manual search of the reference lists of included articles to identify other relevant articles, in accordance with updated methodological guidance for conducting a scoping review [33].

### 2.4. Identification and Selection of Relevant Studies

We uploaded articles to Covidence for selection. When downloads from databases were not in the correct format, articles were uploaded to Mendeley Reference Manager 2.122.0 and converted to the correct format for upload to Covidence. Two reviewers reviewed relevant abstracts (the main author and an independent reviewer), using the predetermined inclusion and exclusion criteria. No date range was established because we wanted the scoping review to consider all sensory integration patterns from the initial development of ASI. When a disagreement arose regarding the inclusion of an abstract, a third (independent) reviewer reviewed the abstract to resolve it. Both primary reviewers then conducted a full-text review for inclusion, and the third reviewer again reviewed the articles to resolve any further disagreement that might occur.

We considered all study designs to enable inclusion of all relevant studies on the subject. The selected studies included quantitative and quantitative studies as well as retrospective record review methodologies, in addition to prospective studies. All articles reviewed involved research conducted within the discipline of occupational therapy, which is unsurprising since children with sensory integration problems are most often assessed and treated by occupational therapists.

### 2.5. Charting the Data

We developed an extraction table in Covidence and extracted all relevant data from the included articles. The data were extracted and charted by the first author. The data were charted and summarized into tables and figures.

## 3. Results

**Figure 1:**
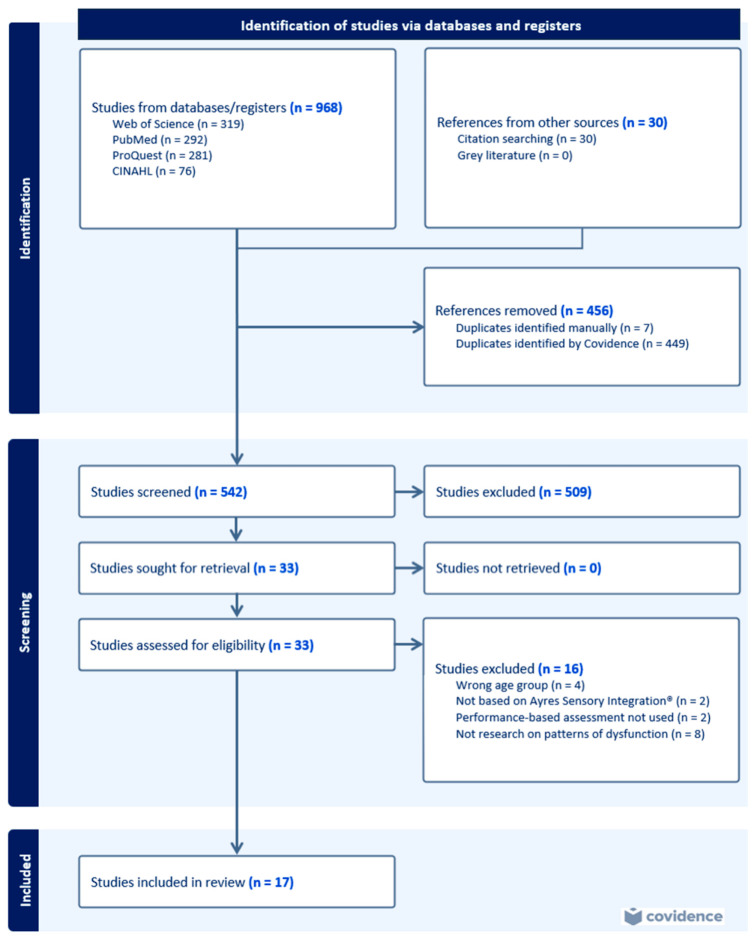
Preferred reporting items for systematic reviews and meta-analysis.

As indicated in the PRISMA flow chart (Figure 1), a search of four databases yielded a total of 968 studies. The other databases did not yield any results. A total of 30 references were also found from other sources, mainly citation searches. This resulted in a total of 998 references imported into Covidence for screening. Of these, 456 duplicates were removed, leaving 542 abstracts; of these, 509 did not meet the eligibility criteria and thus were excluded. We conducted full article screening on the remaining 33 articles, of which 16 were excluded because they did not meet the inclusion criteria (i.e., participants were outside the specified age range, the study did not include a performance-based assessment instrument, or the study was not based on the ASI approach). Seventeen articles remained for data extraction.

### 3.1. Characteristics of the Included Articles

While we found many similarities among the remaining 17 articles, we also noted some variations (Table 1). The 17 articles included in this study were published between 1963 [35]; [S1] and 2023 [8]; [S17]. An examination of publication frequency by decade indicates that more than one quarter of the articles (29.41%) were published between 1960 and 1969 [7,15,35,36,37]; [S1–S5], while an additional 23.53% were published between 2010 and 2019 [5,6,31,38]; [S13–S16]. Only 11.76% of the articles were published between 1990 and 2009 [2,3]; [S11,S12]. The same methodology and geographical location were present in most of the articles, and all studies applied a quantitative design. Nonetheless, the identification of sensory integration patterns was based on heterogeneous analytical strategies, including FA, CA, and mixed-method analytical frameworks, among various other techniques. Factor analysis and/or CA were used in 13 studies (76.47%) [2,3,4,5,6,7,12,15,35,36,37,39,40]; [S1–S5,S7–S13,S15]. Six studies (35.29%) used retrospective record reviews in which de-identified data were extracted and then analyzed [2,3,5,6,31,38]; [S11–S16]. The majority of studies were conducted in the United States (mainly in California), except for one that incorporated data from both the US and Canada [2]; [S11] and one that was conducted in South Africa [5]; [S15]. Two studies (11.76%) had a small sample size of 0–50 participants [37,38]; [S5,S14], with the smallest sample size being 36 participants [37]; [S5]. The largest sample size included 10,475 records from a scoring site in which test scores were collected, reviewed, and analyzed [2]; [S11]. Studies were conducted in various settings, of which the most frequent was schools (six articles; 33.29%) [4,12,15,37,39,41]; [S2,S5–S8,S10], followed by therapy clinics (four articles; 23.53%) [6,31,38,40]; [S13,S16,S14,S9]. In three articles (17.65%), the location of the study was not reported [5,7,36]; [S15,S4,S3].

### 3.2. Assessment Instruments Used in Research Studies

**Figure 2:**
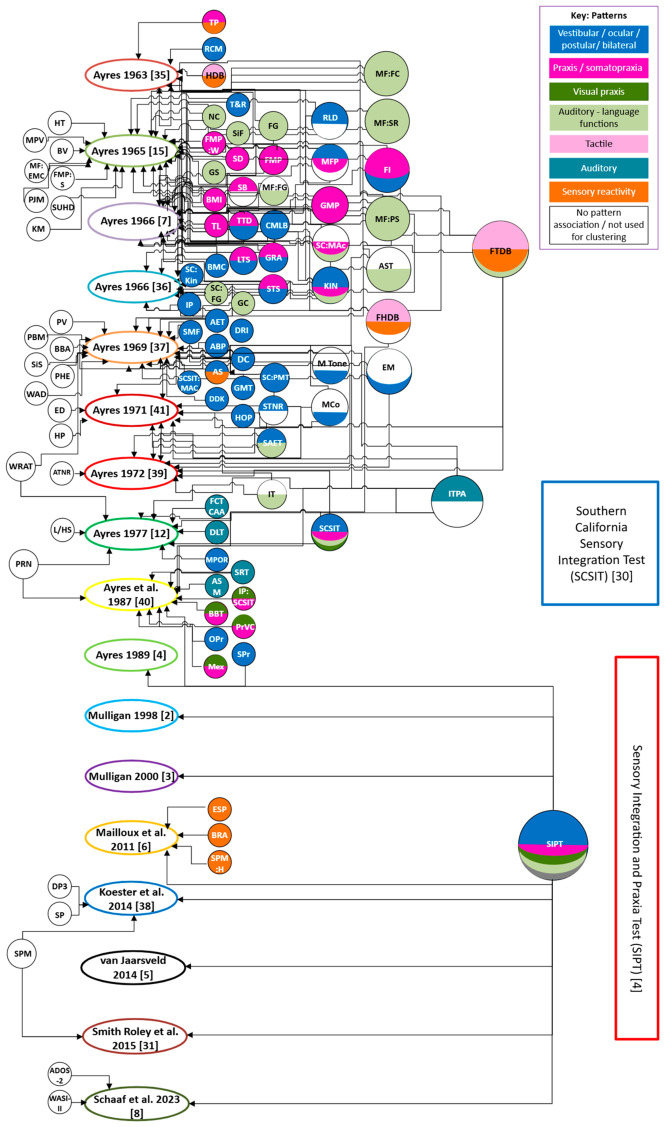
Assessment instruments used in all articles [2,3,4,5,6,7,8,12,15,30,31,35,36,37,38,39,40].

We reviewed the assessment instruments used to collect data to identify sensory integration patterns in the studies (Table 2 and Figure 2), including how frequently each of the assessment instruments or individual tests were applied. For the purposes of this review, a “test” is defined as an individual test designed to evaluate a single component of sensory functioning (e.g., the post-rotary nystagmus test). Multiple such tests were combined to form composite assessment instruments, such as the SCSIT and SIPT. An “assessment instrument” is defined as a standardized measure comprising multiple tests, such as the SIPT, or multiple sections/subtests, including standardized sensory questionnaires (e.g., the Sensory Processing Measure). Figure 2 provides an overview of the tests and assessment instruments used in the studies included in this review. It serves to illustrate three concepts: (1) the chronological development of tests since 1963, (2) the number of individual tests or assessment instruments used in each study, and (3) the sensory integration patterns associated with each test. Figure 2 also presents the color legend for the sensory integration patterns is also presented. The colored circles represent assessment instruments and are coded by their associated sensory integration patterns. Assessment instruments linked to multiple patterns are shown as multi-colored circles. Circle size indicates the frequency with which the corresponding test or assessment instrument was used across studies, ranging from the smallest circles (one study) to the largest (eight studies). The most widely used assessment instrument across all included studies was the SIPT [4], which was used in studies conducted in 1989–2023 and was applied in eight studies (47.06%) [2,3,4,5,6,8,31,38]; [S10–S17]. The SCSIT was administered in three studies (17.65%) conducted in 1972–1987 [12,39,40]; [S7–S9]. As indicated in Table 2, prior to 1972 Ayres used standardized assessment instruments such as the Marianne Frostig Developmental Test of Visual Perception [42] and the Illinois Test of Psycholinguistic Abilities (ITPA) [43]. Furthermore, she developed a series of individual tests [25,26,27,28,29], which were subsequently combined to form the SCSIT.

### 3.3. Reported Sensory Integration Patterns

In all 17 studies, the analyses determined the presence of sensory integration patterns through examination of associations between test scores. The patterns identified in each study can be seen in Table 2. Despite variations across the studies, several recurring patterns emerged, particularly related to praxis, sensory perception, and bilateral integration; these are elaborated in greater detail below. By contrast, patterns of generalized sensory integrative dysfunction, auditory-language functions, and tactile defensiveness were reported less consistently across studies.

Patterns related to praxis were consistently noted. Various names were applied to this pattern over the years depending on the specific assessment instruments, statistical methods used in the studies, and test score groupings. Patterns included “apraxia” [35]; [S1], “developmental apraxia” [15]; [S2], “praxis” [12,36,40]; [S4,S6,S8], “somatosensory and motor planning” [12]; [S8], “general visio-somatic function” [40]; [S9], and “dyspraxia” [2,3]; [S11,S12]. Some variations include “visuo-somatodyspraxia” [5,6,40]; [S10, S13, S15], “dyspraxia on verbal command” [4,31]; [S10,S16], “imitation praxis” [31]; [S16], and “visual praxis” [31]; [S16]. It is noteworthy that numerous studies have consistently demonstrated an early-emerging pattern in which scores on tactile perception tests are associated with scores on praxis tests involving total body imitation or planning of actions.

Patterns related to associations among two or more tests that measure sensory perception were also consistently noted. Again, various names were applied to this pattern over the years, depending on the specific assessment instruments, statistical methods used in the studies, and test score groupings. These patterns were referred to using several names, including “form and position in two-dimensional space,” “perceptual dysfunction: visual figure–ground discrimination” [15,35]; [S1,S2], “visual-perceptual function” [7]; [S4], “form and space perception” [39,41]; [S6,S7], “visual perceptual deficits” [2]; [S11], “tactile and visual discrimination” [6]; [S13], “visual perception & visual motor” [8]; [S17], and “motor-free visual perception” [31]; [S16].

Patterns related to bilateral integration were also commonly found. Ayres identified patterns called “deficit of integration of function of the two sides of the body” [15,35]; [S1,S2] and “interaction of function of two sides of the body with emphasis on tactile perception” [36]; [S3]. Like the other patterns, the names given to the patterns varied depending on the specific assessment instruments, statistical methods, and test score groupings; such names included “postural and bilateral integration” [37,41]; [S5,S6], “bilateral integration and sequencing” [2,3,4,5]; [S10–S12,S15], and “vestibular and proprioceptive bilateral integration and sequencing” [6,38]; [S13,S14]. Finally, Smith Roley et al. described this pattern as “vestibular bilateral integration and sequencing” [31]; [S16].

Several other patterns were noted, though they were found to be less consistent. A pattern termed “generalized sensory integrative dysfunction” [4]; [S10], “generalized practice dysfunction” [2]; [S11], and “generalized sensory integration dysfunction and dyspraxia” [3]; [S12], characterized mostly by many very low scores, was noted in only three studies [2,3,4]; [S10–S12]. Ayres identified an “auditory-language-sequencing function” [37]; [S5], later described as “auditory-language functions” [12,39,41]; [S6–S8]. Finally, several studies have identified a pattern referred to as “tactile defensiveness,” originally described by Ayres [15,35]; [S1,S2] and subsequently described by Mailloux et al. as “tactile defensiveness and attention problems” [6]; [S13]. The remaining patterns were only mentioned in one or two studies.

## 4. Discussion

Ayres sought to understand the underlying sensory integration functions that either supported or impeded children’s successful participation in their occupational roles. To accomplish this, she primarily utilized FA, a statistical technique designed to explain associations among variables and identify latent constructs [14]. When applying this type of analysis, the researcher must consider the possible “factors” that contribute to the association. Ayres’ systematic research program, along with subsequent studies conducted over many decades, has contributed to an understanding of previously unrecognized sensory integration patterns of function and dysfunction. It should be emphasized, however, that the heterogenous analytical strategies used across the different studies to identify these patterns complicate the direct comparability of the patterns.

At the inception of her research, Ayres lacked specific assessment instruments for the evaluation of all the sensory integration difficulties she encountered in her clinical practice. From 1963 to 1971, she administered various tests while developing the SCSIT, published in 1972 [30]. Consisting of 18 tests, the SCSIT provides a comprehensive set of tests of most sensory integration functions [30]. The findings from these studies established an understanding of commonly noted sensory integration patterns. From 1972 to 1988, there was a marked reduction in the use of individual tests in studies, as the standardized SCSIT became the principal set of tests applied in studies. The SIPT, a revised and re-standardized set of 17 tests, was published in 1989 [4,32]. After 1990, the SIPT emerged as the primary assessment instrument, with only four studies incorporating additional assessment instruments [6,8,31,38]; [S13,S14,S16,S17]. In these investigations, additional instruments—such as the Sensory Processing Measure [44] and the Sensory Profile [45]—were used primarily to assess sensory reactivity.

As shown in Table 2, before the publication of the SCSIT [30], studies generally identified up to five sensory integration patterns. Ayres posited that possible sensory integration patterns likely exceeded those known at that juncture [39]. Upon publication of the SCSIT as a set of tests, and the utilization thereof in research, new patterns emerged. The use of the SIPT spurred further research and uncovered new patterns due to test enhancements and new tests added to those from the SCSIT. Certain sensory integration patterns identified in earlier research persisted, albeit with changes over time. It is pertinent to note that the names given to the patterns relate to the types of assessment instruments used and can be affected by the analytical methods used. Consequently, the pattern groupings will vary and will be discussed in greater depth.

### 4.1. Patterns Related to Praxis, Including Visuopraxis and Somatopraxis

Praxis refers to the use of sensory information in the planning and execution of goal-directed tasks by ideating, organizing, and performing a sequence of unfamiliar actions [19,46]. In studies conducted from 1963 to 1971, Ayres applied the terms “apraxia,” “developmental apraxia,” and “praxis,” noting an association between this pattern and tactile functions [7,15,35,37,41]; [S1,S2,S4–S6]. This observation that a link exists between somatosensory perception and motor planning represented an important early milestone in the development of ASI theory [11]. Ayres also introduced the term “somatosensory and motor planning” [12]; [S8]. Later, when the SIPT were included, the terms “praxis” and “dyspraxia” were used [2,3,8]; [S11,S12,S17]. In a study focused on determining various types of praxis, “general visuo-somatopractic function” was identified [40]; [S9]. Other studies identified “visuo- and somatodyspraxia” [4,5] [S10,S13,S15], the latter of which reflects the strong association between the praxis tests and the perception of temporal and spatial qualities of tactile input consistently demonstrated in research findings [40]. Smith Roley et al. identified “visual praxis” and “imitation praxis” [31]; [S16].

Across the studies, praxis consistently exhibits an association with tactile input. Ayres’ early investigations [7,15,37] [S2,S4,S4] established the basis for her understanding of this association between praxis and tactile input [11]. Longitudinal analyses of research findings and the changes in terminologies associated with praxis suggest that Ayres progressively recognized the sensory foundations of praxis, particularly from the year 1977 onwards. Therefore, terminologies adopted included “somato-praxis” and “visuo-praxis.” A strong association between visuodyspraxia and somatodyspraxia has been reported in multiple investigations [3,4,6] [S10,S12,S13] in which a combined pattern of visuo-somatodyspraxia was identified [11]. Praxis problems impact participation in daily activities, including social interactions, play, and participation in self-care activities [11,47].

### 4.2. Patterns Related to Bilateral Integration and Sequencing/Ocular/Postural Function

A relationship between postural and ocular function, vestibular function, bilateral control, and laterality has long been recognized based on neural functions [17]. Between 1963 and 1971, studies frequently identified some of these functions as a “bilateral integration” pattern named “deficits of integration of function of the two sides of the body” or “postural and bilateral integration,” noting an association between tests of those functions and observations of avoidance of crossing the body midline and difficulties distinguishing the two sides of the body [15,35,36,37,41]; [S1–S3,S5,S6]. Two studies identified “postural and ocular mechanisms/reactions” as distinct patterns, through numerous individual tests were administered on children with diagnosed learning disabilities [12,39]; [S7,S8]. Later, the term “bilateral integration and sequencing (BIS)” [2,3,4,5] [S10–S12,S15] was applied. Ayres observed that when comparing a cohort of children exhibiting learning or sensory integration challenges with a normative cohort, the BIS pattern was absent in the normative cohort [4]; [S10]. The finding of an absent BIS pattern in the normative cohort led Ayres to believe that the vestibular-based inefficiencies thought to underlie the BIS pattern would not be present at any point during typical development [48]. The BIS pattern was sometimes called “vestibular and proprioceptive bilateral integration and sequencing” [6,38]; [S13,S14] or “vestibular bilateral integration and sequencing” [31]; [S16]. Although Ayres hypothesized that vestibular functions were associated with this pattern, the post-rotary nystagmus test (PRN)—i.e., the main test for vestibular functions—presented confounding results as both high and low scores on this test were atypical [4]. As a result, high and low scores present in a clinical sample would frequently “cancel each other out” in statistical analyses. However, when analyses included only atypically low scores in the analyses, the association with vestibular function became evident and, accordingly, the name given to the pattern reflected this association (e.g., “vestibular and proprioceptive bilateral integration and sequencing”). Children with atypically low PRN scores were found to also have poorer scores on SIPT items measuring vestibular function than children with average or atypically high PRN scores [49]. Recently, this pattern has been denoted as “vestibular-proprioceptive and bilateral motor skill” [8]; [S17]. Children exhibiting difficulties within the vestibular bilateral integration and sequencing pattern are frequently reported to have problems in school related to reading and writing [11].

### 4.3. Visual Sensory Perception

Studies conducted from 1963 to 1971 sometimes identified patterns related to perceptual dysfunction, which were mostly related to visual perception. Patterns included “perceptual dysfunction: visual figure-ground discrimination,” “form and space perception,” and “form and position in two-dimensional space” [7,15,35,41]; [S1,S2,S4,S6], as well as “visual perceptual deficit” [2]; [S11], “motor-free visual perception” [31]; [S16], and “visual perception & visual motor” [8]; [S17]. Moreover, a pattern showing a relationship between “tactile and visual discrimination” was noted [5,6]; [S13,S15]. Contemporary neuroscience research shows that the superior colliculus in the midbrain integrates visual, auditory, and somatosensory inputs and integrates them with motor output generating goal-directed behaviors [50].

### 4.4. Auditory-Language Related Functions

Ayres identified the pattern called “auditory-language functions” [41]; [S6], which subsequently appeared in only two studies [12,39]; [S7,S8]. The emergence of this pattern may be due to the inclusion of the Illinois Test of Psycholinguistic Abilities, which includes assessment of auditory functions [12,39,40]. “Dyspraxia on verbal command” was also observed [4,31]; [S10,S16]. Ayres noted that the pattern of “Dyspraxia on Verbal Command” reported in the SIPT manual generally links to impairment of linguistic components [4] (p. 145).

### 4.5. Tactile Defensiveness/Sensory Reactivity

Ayres identified the pattern of tactile defensiveness that was concomitant with distractible and hyperactive behavior [15,35,36]; [S1–S3]. In one study, she used observations to identify tactile defensiveness [35]; [S1]. In later studies, she applied a non-standardized rating scale to identify tactile defensiveness [15,36]; [S2,S3]. Since no standardized assessments for sensory reactivity existed during her lifetime, she included observations and non-standardized questionnaires in her studies. However, after the 1970s, she no longer included these measures, primarily in response to critiques of her use of non-standardized assessment instruments. In 1999, standardized questionnaires designed to assess tactile defensiveness (and overall sensory reactivity) became available [44,45,51,52,53,54]. Using the Sensory Processing Measure (SPM) to collect data about sensory reactivity, Mailloux et al. identified a pattern they termed “tactile defensiveness and attention problems” [6]; [S13]. With the publication of the Diagnostic and Statistical Manual of Mental Disorders, Fifth Edition (DSM-5) [55], in which sensory features were incorporated into the diagnostic criteria for autism, a shift in terminology occurred. The DSM-5 specifically describes hyper- or hypo-reactivity to sensory input [55]. Mailloux & Miller-Kuhaneck suggest occupational therapists consider studying sensory reactivity with enhanced precision [56]. This requires assessment instruments with strong specificity and reliability. Children experiencing sensory reactivity problems often have reported difficulties with behavior and attention [11].

### 4.6. Generalized Sensory Integrative Dysfunction

A pattern named “generalized sensory integrative dysfunction” was noted in three studies [2,3,4]; [S10–S12] that applied CA in contrast to FA. The pattern name varied, including the terms “generalized practic dysfunction” [2]; [S11] and “generalized sensory integration dysfunction and dyspraxia” [3]; [S12]. The main feature of this grouping was very low scores on all tests and thus did not lead to hypotheses about the underlying “factor” associating these test scores.

### 4.7. Neural Mechanisms Underlying Sensory Integration Patterns

Given that ASI is grounded in the neurosciences, it is important to examine the neural mechanisms underlying sensory integration and associated sensory integration patterns. Sensory information is conveyed from peripheral receptors to the central nervous system and is projected to multiple brain regions, each processing input differently to support distinct functional outcomes. Using continuous sensory feedback, the cerebellum coordinates and times movement, detects and corrects errors, and adjusts motor actions [50]. The basal ganglia select, initiate, and activate appropriate motor programs and are critical for procedural memory and motor learning [50]. Tactile input to the insular cortex, which projects to the orbitofrontal cortex, is central to homeostatic regulation, interoception, and affective experiences [19]. Ayres proposed that the limbic system is a primary locus for difficulties in registration [57]. The emotion-related structures, including the amygdala and insula, detect and evaluate salient information [20,58]. In this context, the insula is a core node of the salience network, responding to relevant and novel sensory stimuli [20,58]. The salience network mediates dynamic switching between default mode networks and task-based networks, thereby supporting cognitive control [20,58]. In addition, the insula operates as an integration hub for emotional and physiological perception and is essential for attention [20]. The thalamus serves as an integrative hub through its reciprocal connections with the cortex and mediates interactions between cortical and subcortical systems [50]. Except for olfactory input, all sensory information passes through thalamic relay centers before reaching the sensory cortex.

Somatosensation comprises tactile and proprioceptive input transmitted to the cortex via the thalamus in the dorsal column–medial lemniscal pathway [19,20]. Tactile inputs reach the posterior parietal cortex, where they are integrated with visual information and motor signals [19]. Multisensory integration of somatosensory, vestibular, and visual information occurs at multiple levels in the central nervous system, including the vestibular nuclei, the thalamus, and the cortex [59]. This integration of multisensory inputs is essential for detecting postural stability, self-motion, and spatial orientation [19]. Collectively, these findings support the presence of close connections between somatosensation and other sensory systems as originally proposed by Ayres [19]. Ayres further hypothesized that the somatosensory system is pivotal for praxis. This hypothesis was supported by empirical studies using FA and CA, which consistently demonstrated a clear relationship between tests of tactile perception and praxis [4,7,12,36,40]; [S3,S5,S6,S8,S10]. As early as 1961, Ayres emphasized body schema as the foundation for praxis and its relationship with somatosensation [60]. Based on this proposed relationship, she identified core intervention principles for dyspraxia, emphasizing engagement in active, sensory–motor activities that elicit adaptive motor responses calibrated to a “just-right level” of challenge [19,26]. More recent neuroscientific evidence indicates that somatosensory input influences feedforward processing and prediction of movement [19]. Contemporary findings also identify the cerebellum as a key subcortical hub for sensorimotor integration, potentially by expanding the range of sensorimotor association [50]. The cerebellum adjusts the timing and intensity of muscle activations to improve new motor actions; thus, motor learning depends on its capacity to map outgoing motor commands to the anticipated sensory feedback from the movements [50]. These findings further support Ayres’s original hypothesis that somatosensation is central to praxis.

Ayres initially proposed that the vestibular system strongly affects brain and behavioral functions [61];. Vestibular input travels from the brainstem to multiple cortical regions, including the insula, parietal operculum, temporo-parietal junction, superior temporal gyrus, hippocampal and para hippocampal cortices, somatosensory cortex, and mid-cingulate cortex [19,62]. Vestibular projections to the cerebellum refine head and postural control, forming a basis for the development of complex motor skills [19]. Furthermore, the vestibular system contributes to bilateral motor coordination as it is an inherently bilateral system influencing muscle activation throughout the body [19]. A sensory integration pattern called “deficits in vestibular-bilateral-integration” [6,12,38,40]; [S6–S8,S13] was identified due to reduced vestibular responses during the post-rotary nystagmus test. This pattern has been associated with poor bilateral integration, decreased coordination of eye and head movement, reduced prone extension, and poor equilibrium reactions [19]. Contemporary neuroscience supports Ayres’ original hypothesis demonstrating that vestibular information is responsible for essential functions including postural control, balance and equilibrium, regulation of arousal, bilateral coordination, visual field stability, and spatial perception [19].

### 4.8. Included Articles

A total of 17 articles were included in this scoping review. The methodologies used in all studies were homogeneous with all studies utilizing a quantitative method: Six studies conducted retrospective record reviews, and only one conducted a non-randomized experimental study. This is unsurprising as quantitative data analysis is key for researching sensory integration patterns. Most of these studies (76.47%) used FA and/or CA, and one used stepwise regression equations [41]; [S6]. Some authors have argued that FA is preferable to regression techniques for this type of analysis [63].

These studies span 60 years, from 1963 to 2023; a significant proportion (58.82%) were published during an initial 28-year period from 1963 to 1989, during Ayres’s lifetime. By contrast, only seven studies (41.18%) were published over the subsequent 33 years from 1990 to 2023. The absence of studies from 1990 may be attributed to the publication of the SIPT in 1989 [4], the last comprehensively developed ASI assessment instrument before the introduction of the EASI [32]. Research on the sensory integration patterns as identified by the SIPT was aimed at both verifying the patterns [3,6] [S12,S13] and determining patterns among children from varied diagnostic groups or different populations [5,8,31,38]; [S14–S17]. A new ASI assessment instrument, the EASI, was recently developed and is currently undergoing refinement [32]. Published articles for the EASI cover age trends and internal consistency, construct validity and internal reliability, cultural adaptations, and a case report [64,65,66,67,68]. However, investigations on sensory integration patterns remain unpublished.

### 4.9. Future Research and Limitations

Since 2018, several articles on the development of the EASI have been published. Designed as a practical, affordable, and electronically accessible assessment instrument for the evaluation of sensory integration, as well as an open-access assessment, the EASI will facilitate ongoing research [32]. Comparison of sensory integration patterns identified by the EASI with those in this scoping review is yet to be performed.

There is a need to further explore the relationship between the sensory integration patterns of dysfunction and their impact on participation in occupations.

Inclusion of articles on sensory integration patterns in different diagnostic groups is a limitation that can influence conclusions regarding the patterns found over time. These patterns differ from those in children with sensory integration difficulties without additional diagnoses. Although this scoping review provides a detailed description of the patterns, the nature of this scoping review means no studies were excluded based on methodology or population size. This may affect the results as it is based on studies with limited generalizability.

### 4.10. Implications for Occupational Therapy Research and Practice

Best practice evaluation and intervention in occupational therapy based on the ASI approach rely on comprehensive research of the assessment instruments and interpretation of their results. This scoping review demonstrates extensive research conducted on development of assessment instruments and sensory integration patterns for interpretation. Differences in patterns across locations and diagnostic populations were noted.

This scoping review has the following implications for occupational therapy practice.

- This study provides a comprehensive overview of sensory integration patterns in ASI over time, confirming it is rooted in rigorous research.
- Occupational therapy practitioners using the ASI approach should stay updated on research developments, as sensory integration patterns exhibit variations with new studies.
- Despite the development and refinement of the EASI, there are currently only sensory integration patterns available based on SIPT data, underpinned by extensive research.

## 5. Conclusions

This review indicates that several patterns of sensory integration function and dysfunction have been consistently demonstrated through research, including studies in various diagnostic groups. Although some variations to patterns can be seen due to the specific assessment instruments and statistical methods applied, van Jaarsveld highlighted consistent patterns across different contexts [5]. She suggested that future research prioritize the development of appropriate and cost-effective assessments applicable to the Global South, where many children lack access to expensive services, rather than focusing on patterns that appear consistent [5]. Since 2014, only one relevant study has been published, specifically on patterns in a different diagnostic group. The ASI approach is based on decades of research focusing on the development of reliable assessment instruments and patterns. Although a comprehensive examination of the implications of the use of different statistical methodologies across studies was beyond the scope of the present review, this scoping review nonetheless improves understanding of the historical development of the field and helps advance future research.

## Figures and Tables

**Table 1:** Characteristics of the studies included in the scoping review.

Study/Article Characteristics	N = 17 *n* (%)	Study/Article Characteristics	N = 17 *n* (%)
Types of information source		Study setting	
Primary research article	16 (94.12)	Not available	3 (17.65)
Test manual	1 (5.88)	Private clinic/practice	4 (23.53)
Study Locations		Schools	6 (35.29)
United States of America (USA)	15 (88.24)	University of Southern California	1 (5.88)
USA & Canada	1 (5.88)	Western Psychological Services	2 (11.76)
South Africa	1 (5.88)	Part of a larger study	1 (5.88)
Publication dates		Sample sizes	
1960–1969	5 (29.41)	0–50	2 (11.76)
1970–1979	3 (17.65)	51–100	5 (29.41)
1980–1989	2 (11.76)	101–200	5 (29.41)
1990–1999	1 (5.88)	201–300	3 (17.65)
2000–2009	1 (5.88)	301–400	0 (0.00)
2010–2019	4 (23.53)	401–500	0 (0.00)
2020–2024	1 (5.88)	501–1000	0 (0.00)
		>1000	2 (11.76)
Methodology used		Methods of data analysis	
Quantitative	17 (100)	Factor analysis	11 (64.71)
Qualitative	0 (0.00)	Factor and cluster analysis	1 (5.88)
Retrospective record review	6 (35.29)	Cluster analysis	1 (5.88)
Non-randomized experimental study	1 (5.88)	Stepwise regression	1 (5.88)
		One sample *t*-test	1 (5.88)
		Correlation coefficient	2 (11.76)

**Table 2:** Development of sensory integration patterns.

Author	Year	Article Number	Participant:			Tests/Assessment Instruments Used	Data Analysis	Patterns Identified
			Age	Condition	Sample Size			
Ayres [35]	1963	S1	6 yrs–7 yrs	Learning or behavioral difficulties	100	Motor planning; assuming demonstrated postures; ability to manipulate objects; finger identification; tactile perception: localize stimulus; discriminate between one and two stimuli; perceive two simultaneous stimuli; identify figures drawn on cheek and hand; manual perception of form; kinesthesia; Frostig’s test of form constancy; position in space and relations; figure ground perception; right–left discrimination; reluctancy to cross the midline; hyperactive, distractible behavior.	Factor analysis	Apraxia Perceptual dysfunction: form and position in space Deficit of integration of function of the two sides of the body Perceptual dysfunction: visual figure–ground Tactile defensiveness
Ayres [15]	1965	S2	5 yrs 9 mths–8 yrs 0 mths	Typical developing children and learning difficulties	150	Southern California (SC) motor accuracy test; Graphic skill; kinesthetic memory; localization of tactile stimuli; eye pursuit; skin designs; manual perception of form; standing balance: eyes open and closed; visual perception of verticality. Marianne Frostig (MF): Eye–motor coordination; figure–ground; position in space; spatial relations. Ayres Space Test (AST); hands test; motor planning—gross; right–left discrimination; strength of unilateral hand dominance; degree of agreement between eye and hand dominance; body visualization; crossing the mid-line of the body; perception of joint movement; fine motor planning: wire–grommet device and string winding; two-point tactile discrimination; two simultaneous tactile stimuli; superimposed figures; Gestalt completion; time and rhythm; number concepts; freedom from tactile defensive behavior; freedom from hyperactive and distractible behavior.	Factor analysis	Developmental apraxia Perceptual dysfunction: form and position in two-dimensional space Tactile defensiveness Deficits of integration of function of the two sides of the body Perceptual dysfunction: visual figure–ground discrimination
Ayres [36]	1966	S3	4 yrs–8 yrs	Typical developing children	64	MF: Figure ground; from constancy; position in space; spatial relations; SC kinesthesia and tactile perception tests (manual form perception; finger identification; graphesthesia; localization of tactile stimuli; perception of double tactile stimuli; imitation of postures); SC figure–ground visual perception test; SC motor accuracy test; AST; motor planning; bilateral motor coordination; crossing the mid-line of the body.	Factor analysis	Visual–motor ability Interaction of function of two sides of the body with emphasis on tactile perception Tactile defensiveness
Ayres [7]	1966	S4	4 yrs–8 yrs	Typical development/Probable central nervous system dysfunction/Cerebral palsy	92	SC motor accuracy test; MF: Form constancy; position in space; spatial relations; AST; bilateral motor integration; finger identification; graphesthesia; kinesthesia; standing balance; fine motor planning; simultaneous tactile stimulation; tactile localization; gross motor planning; right–left discrimination; visual figure–ground perception; freedom from hyperactive and distractible behavior; freedom from tactile defensive behavior.	Factor analysis	Praxis Visual–perceptual function
Ayres [37]	1969	S5	6 yrs 0 mths–9 yrs 10 mths	Learning difficulties	36	Muscle tone; muscle co-contraction; arm extension test (AET); passive head extension; arched back posture; postural background movements; hopping; perception of verticality; diadokokinesia; SC perceptual–motor tests; non-dominant hand sc motor accuracy compared to dominant hand SC motor accuracy; Illinois Test of Psycholinguistic Abilities (ITPA); Birch–Belmont Auditory–Visual Integration; Wepman Auditory Discrimination; discrimination of reversed images; design copying; sequence in space; eye movements; sensorimotor functions; freedom from tactile defensiveness; freedom from hyperactive and distractible behavior; attention span; Gates–MacGinitie Test; intelligence.	Factor analysis	Auditory-language-sequencing function Postural and bilateral integration Praxis (tactile motor planning).
Ayres [41]	1971	S6	5 yrs 8 mths–11 yrs 0 mths	Learning difficulties	148	SC Battery of Perceptual–Motor Tests; ITPA; hand preference; eye dominance; muscle tone; Schilder’s Arm Extension Test; co-contraction; eye movements across the mid-line; asymmetrical tonic neck reflex (ATNR); symmetrical tonic neck reflex (STNR); intelligence.	Stepwise regression	Postural and bilateral integration Praxis Functions of the left side of the body (right hemisphere dysfunction) Form and space perception Auditory-language functions
Ayres [39]	1972	S7	7 yrs 4 mths–10 yrs 0 mths	Learning difficulties	148	Freedom from hyperactivity and distractibility; freedom from tactile defensiveness; muscle tone; Schilder’s arm extension test; co-contraction; eye movements; ATNR; STNR; Southern California Sensory Integration Test (SCSIT); ITPA.	Factor analysis	Form and space perception Auditory language functions and intelligence Postural and ocular mechanisms Motor planning, hyperactivity, and tactile defensiveness Reading, with spelling and intelligence closely associated Hyperactivity and tactile perception
Ayres [12]	1977	S8	6 yrs–10 yrs	Learning difficulties	128	SCSIT; Southern California post-rotary nystagmus test (SCPNT); intelligence test (WISC or Stanford–Binet); measurement of postural and ocular responses; indices of lateralization or hemispheric specialization. Portions of the ITPA; Flowers–Costello Tests of Central Auditory Abilities (FCTCAA); wide range achievement test (WRAT); dichotic listening test.	Factor analysis	Auditory-language functions Postural–ocular reactions Eye–hand coordination Somatosensory and motor planning or praxis
Ayres et al. [40]	1987	S9	4 yrs 0 mths–9 yrs 11 mths	Learning and behavioral difficulties	182	Manual expression (MEx) subtest of the ITPA; imitation of postures test of the SCISIT; sequencing praxis test (SPr); praxis on verbal command test (PrVC); oral praxis test (OPr); block building test (BB); SCISIT; SCPRN; auditory sequential memory (ASM) subtest of the ITPA; sentence repetition test (SRT).	Factor analysis	General visuo-somatopratic function with elements linked by concept formation.
Ayres [4]	1989	S10	5 yrs 0 mths–8 yrs 11 mths	Typical developing, learning difficulties, and sensory integration difficulties	293	Sensory Integration and Praxis Test (SIPT).	Factor analysis & cluster analysis	Factor patterns (factor analysis) Bilateral integration and sequencing Somatopraxis Visuopraxis Low-average sensory integration and praxis Dyspraxia on verbal demand High average sensory integration and praxis Cluster patterns (cluster analysis) Low-average bilateral integration and sequencing Generalized sensory integrative dysfunction Visuo-and somatodyspraxia Low-average sensory integration and praxis Dyspraxia on verbal command High average sensory integration and praxis
Mulligan [2]	1998	S11	4 yrs 0 mths–8 yrs 11 mths	Learning difficulties, behavioral difficulties, and mild motor difficulties	10,475	SIPT	Factor analysis	First-order factors Visual perceptual deficit Bilateral integration & sequencing deficit Dyspraxia Somatosensory deficit Second-order factor Generalized praxis dysfunction
Mulligan [3]	2000	S12	4 yrs 0 mths–8 yrs 11 mths	Typical developing or received special education	1961	SIPT	Cluster analysis	Generalized sensory integration dysfunction and dyspraxia-severe Dyspraxia Generalized sensory integration dysfunction and dyspraxia-moderate Low-average bilateral integration and sequencing Average Sensory Integration and Praxis
Mailloux et al. [6]	2011	S13	4 yrs 0 mths–9 yrs 0 mths	Learning difficulties, sensory integration difficulties, attention deficit disorder or speech–language disorders	273	SIPT; Sensory Processing Measure (SPM) Home Form; evaluation of sensory processing (ESP); behavior rating of attention.	Factor analysis	Visuo- and somatodyspraxia Vestibular and proprioceptive bilateral integration and sequencing Tactile and visual discrimination Tactile defensiveness and attention problems
Koester et al. [38]	2014	S14	4 yrs 0 mths–8 yrs 11 mths	Mild to severe hearing loss	49	SIPT (except for the praxis on verbal command test); Developmental Profile 3; Sensory Profile (SP) or SPM.	One sample *t*-test	Characteristics of the vestibular and proprioceptive bilateral integration and sequencing (VPBIS) pattern of sensory integrative dysfunction
Van Jaarsveld [5]	2014	S15	4 yrs 0 mths–8 yrs 11 mths	Sensory integration difficulties	236	SIPT	Factor analysis	Visuo- and somatodyspraxia Bilateral integration and sequencing Tactile and visual discrimination dysfunction
Smith Roley et al. [31]	2015	S16	4 yrs 0 mths–8 yrs 11 mths	Autism Spectrum Disorder	89	SIPT; SPM.	Correlation coefficient	Motor-free visual perception Visual praxis Imitation praxis Vestibular bilateral integration and sequencing Somatosensory: tactile and kinaesthesia Praxis on verbal command
Schaaf et al. [8]	2023	S17	6 yrs 0 mths–9 yrs 6 mths	Autism Spectrum Disorder	93	SIPT; Autism Diagnostic Observation Schedule, Second Edition (ADOS-2); Wechsler Abbreviated Scale of Intelligence—Second Edition (WASI-II).	Correlation coefficient	Praxis Tactile perception Vestibular and proprioceptive functions and bilateral motor skills Visual perception and visual motor

## Data Availability

No new data were created or analyzed in this study. Data sharing is not applicable to this article.

## Supplementary Materials

The following supporting information can be downloaded at https://www.mdpi.com/article/10.3390/brainsci16070700/s1, Table S1. Preferred Reporting Items for Systematic reviews and Meta-Analyses extension for Scoping Reviews (PRISMA-ScR) Checklist [34].

## Abbreviations

The following abbreviations are used in this manuscript:

ASI	Ayres Sensory Integration^®^
FA	Factor analysis
CA	Cluster analysis
SCSIT	Southern California Sensory Integration Tests
SIPT	Sensory Integration and Praxis Tests
EASI	Evaluation of Ayres Sensory Integration^®^
PRISMA-ScR	Preferred Reporting Items for Systematic Reviews and Meta-Analyses Extension for Scoping Reviews
BIS	Bilateral integration and sequencing
PRN	Post-rotary nystagmus test
DSM-5	Diagnostic and Statistical Manual of Mental Disorders, Fifth Edition
